# Bilateral Greater Trochanteric Avulsion Fractures after Bilateral Simultaneous Total Hip Arthroplasty

**DOI:** 10.1155/2018/6596135

**Published:** 2018-04-29

**Authors:** Hiroaki Tagomori, Nobuhiro Kaku, Tomonori Tabata, Hiroshi Tsumura

**Affiliations:** Department of Orthopaedic Surgery, Oita University, Oita, Japan

## Abstract

We report a case of bilateral spontaneous greater trochanteric fracture after bilateral simultaneous total hip arthroplasty (THA) performed via the posterolateral approach during the early postoperative phase. A 75-year-old woman underwent bilateral simultaneous THA (BS-THA) for severe osteoarthritis with developmental dysplasia of the hip; she also presented a limited range of adduction. BS-THA was successful without any intraoperative complications. Rehabilitation with full weight-bearing exercises was initiated the day after the surgery. On the 14th postoperative day, she experienced a spontaneous left greater trochanteric fracture during a walking exercise without any trauma. Osteosynthesis was performed for the fracture on the 18th postoperative day. On the 20th postoperative day, a right spontaneous greater trochanteric avulsion fracture occurred during a transfer exercise without any trauma; this was treated on the 27th postoperative day. In the 18th postoperative month, although the right fragment showed slight upper migration, the patient had no complaints of coxalgia and both hip joints showed an excellent range of motion.

## 1. Introduction

Bilateral simultaneous total hip arthroplasty (BS-THA) yields clinical results comparable to those of two-staged procedures. BS-THA has several advantages such as a decrease in patient anxiety, total treatment cost, and hospitalization period; the use of a single, short anesthetic exposure; and achievement of a postoperative hip flexion angle [1]. Greater trochanteric fractures are one of the rare complications of THA, with an incidence of about 5%. Because greater trochanteric fractures occur due to intraoperative manipulations, they are frequently reported after THA that is performed via an anterior approach [2]. We present a rare case of bilateral greater trochanteric fracture after BS-THA that was performed via a posterolateral approach.

## 2. Case Presentation

A 75-year-old woman presented to our institution after 10 years of conservative treatment for hip osteoarthritis secondary to developmental dysplasia. The patient characteristics were as follows: height, 165 cm; body weight, 50.6 kg; and body mass index, 18 kg/m^2^. Physical examination revealed an antalgic gait while ambulating with a cane. Tenderness was observed in both Scarpa triangles, and the Patrick test was positive for both legs. Her range of motion was moderately restricted, with hip flexion of 110°, extension of 5°, abduction of 30°, adduction of 10°, internal rotation of 10°, and external rotation of 30°. The Harris Hip Score was 52/51 (right/left (Rt/Lt)) points. The bone mineral density (BMD) of the femoral neck was 0.758/0.690 (Rt/Lt) g/cm^2^ and of the lumbar spine was 0.857 g/cm^2^. Although the BMD of the femoral neck (Rt/Lt) and the lumbar vertebra was more than 80% of the mean values in young adults, the patient's *T*-score was low.

**Figure 1:**
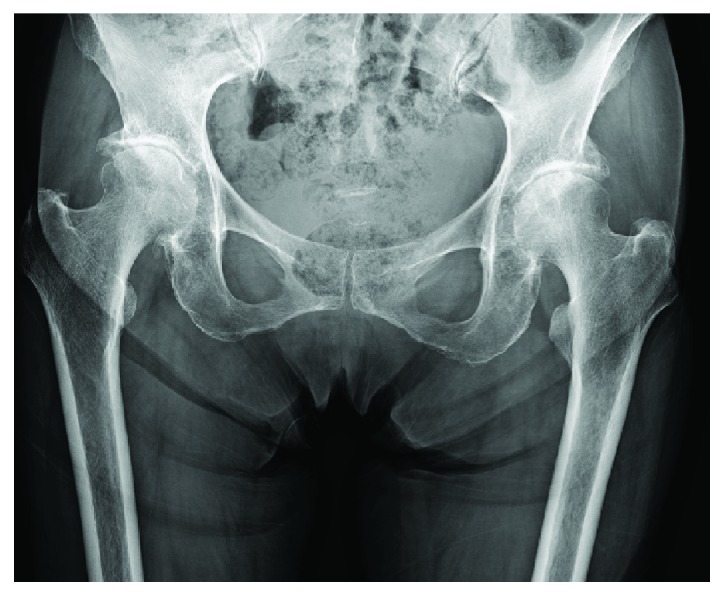
Preoperative radiograph showing osteoarthritis secondary to developmental dysplasia of the hips.

**Figure 2:**
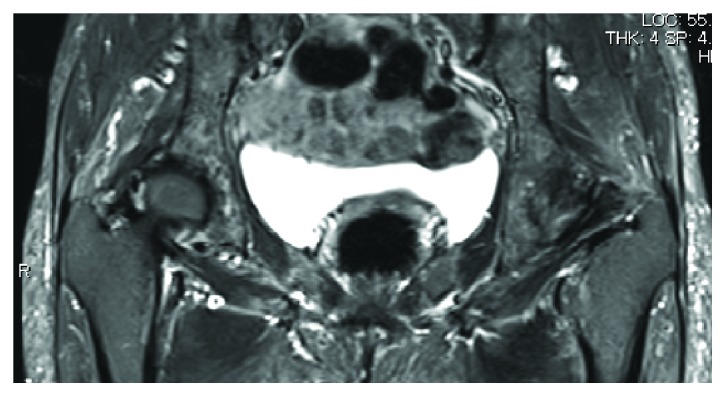
Preoperative magnetic resonance imaging. No abnormal findings are observed.

Plain radiography indicated osteoarthritic changes, represented by hip joint narrowing, osteosclerotic changes in the subchondral bone, and osteophyte formation (Figure 1). However, preoperative magnetic resonance imaging did not show any abnormal findings of the gluteus medius muscle (Figure 2).

The patient underwent BS-THA via a posterolateral approach, which was successful without intraoperative complications. We implanted acetabular cups with a computed tomography-based navigation system (VectorVision Compact Hip CT version 3.5.2; BrainLab, Munich, Germany). The implants included cementless hydroxyapatite-coated acetabular SQRUM cups (Kyocera Medical Co. Ltd., Osaka, Japan), polyethylene acetabular Aquala liners (Kyocera Medical Co. Ltd., Osaka, Japan), and tapered-wedge cementless hydroxyapatite-coated femoral J-Taper stems (Kyocera Medical Co. Ltd., Osaka, Japan). We detached the piriformis, short rotator muscles, and joint capsule from the femur during the posterolateral approach, created a few holes vertically in the intertrochanteric posterior crest before closing the surgical wound, and then attached the piriformis, short rotator muscles, and joint capsule to the intertrochanteric crest with sutures. The total operation time was 5 hours and 57 minutes with an estimated blood loss of 530 mL.

**Figure 3:**
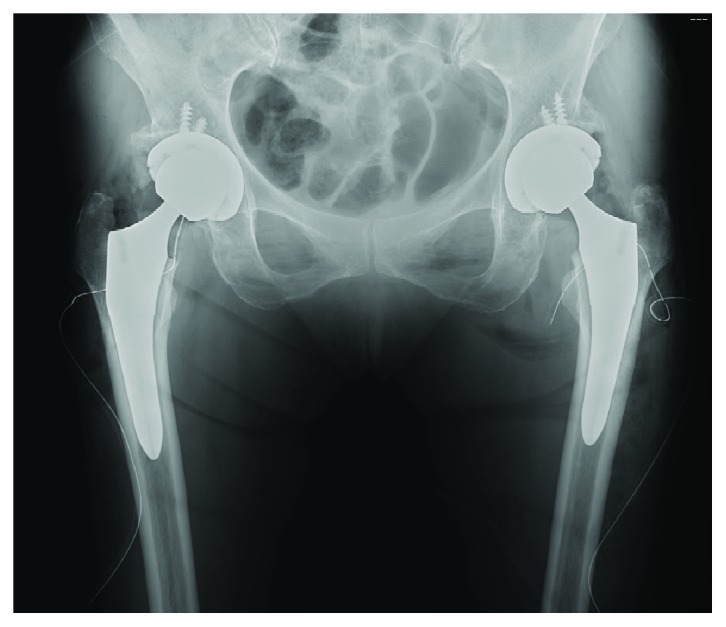
The patient underwent BS-THA via a posterolateral approach, which was successful without intraoperative complications.

**Figure 4:**
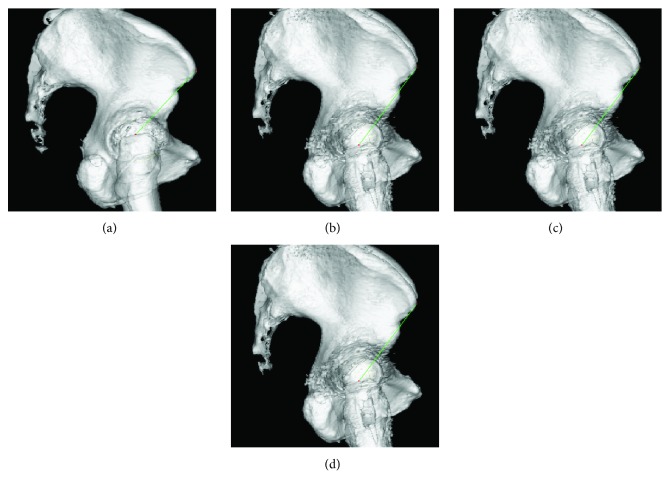
Measurement of the preoperative and postoperative distance from the anterior superior iliac spine to the greater trochanter tip by using the ZedHip system. (a) Preoperative, right. (b) Postoperative, right. (c) Preoperative, left. (d) Postoperative, left.

Postoperative radiographic evaluation demonstrated that the patient's right and left limbs were extended by 14 and 8 mm, respectively, when compared with the preoperative length (Figure 3). We measured the preoperative and postoperative distance from the anterior superior iliac spine to the greater trochanter tip using the ZedHip system (ZedHip Lexi Co. Ltd., Tokyo, Japan) (Figure 4).

**Figure 5:**
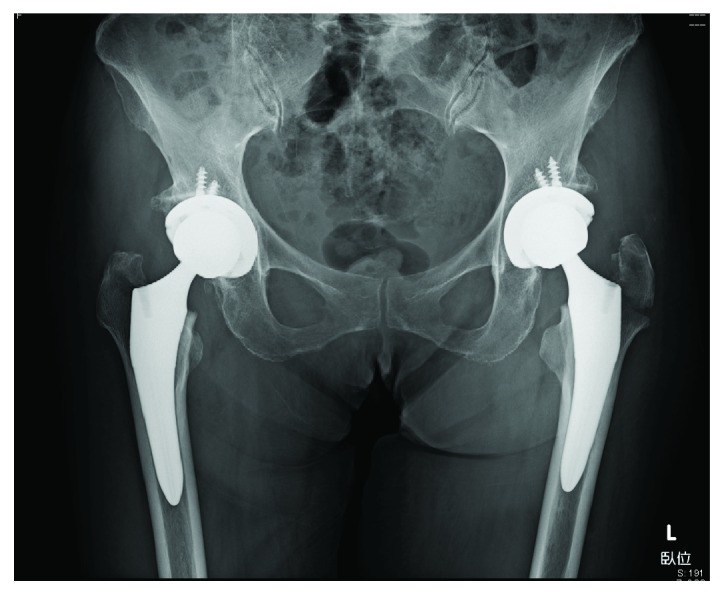
On the 14th postoperative day, the patient complained of left coxalgia during a walking exercise without any falls. Plain radiographs revealed a left greater trochanteric avulsion fracture.

**Figure 6:**
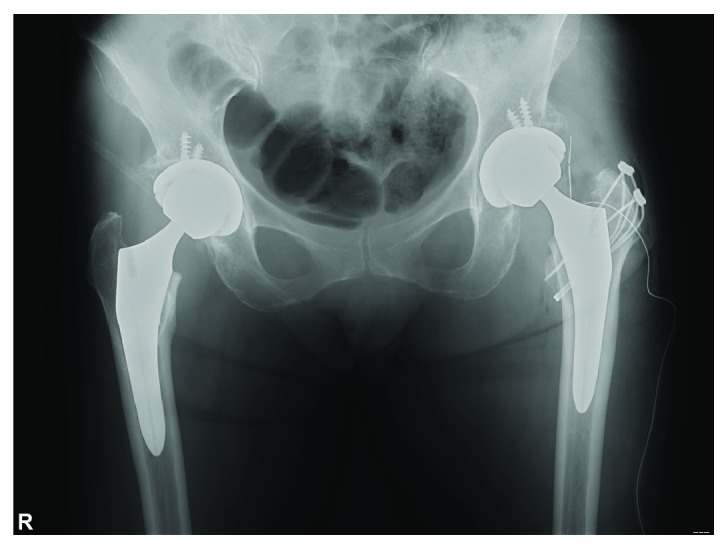
The fracture was fixed using tension band wiring on the 18th postoperative day.

**Figure 7:**
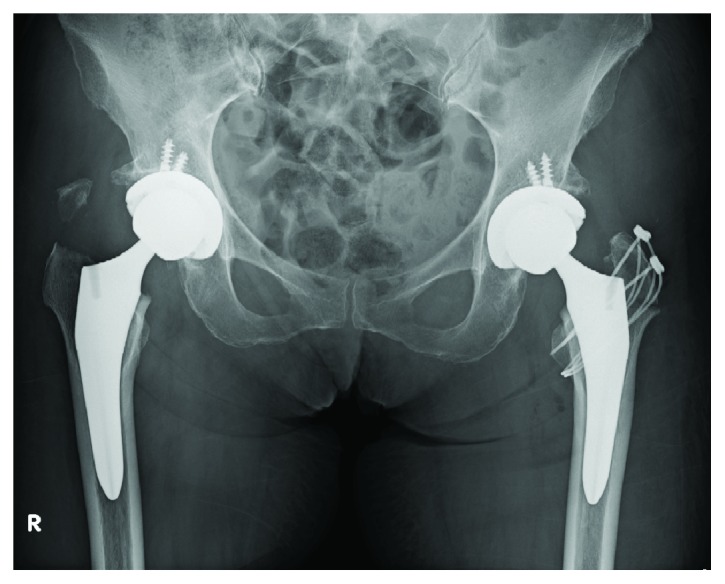
On the 20th postoperative day, right coxalgia emerged during a transfer exercise. The greater trochanteric avulsion fracture on the right side was detected on plain radiographs.

**Figure 8:**
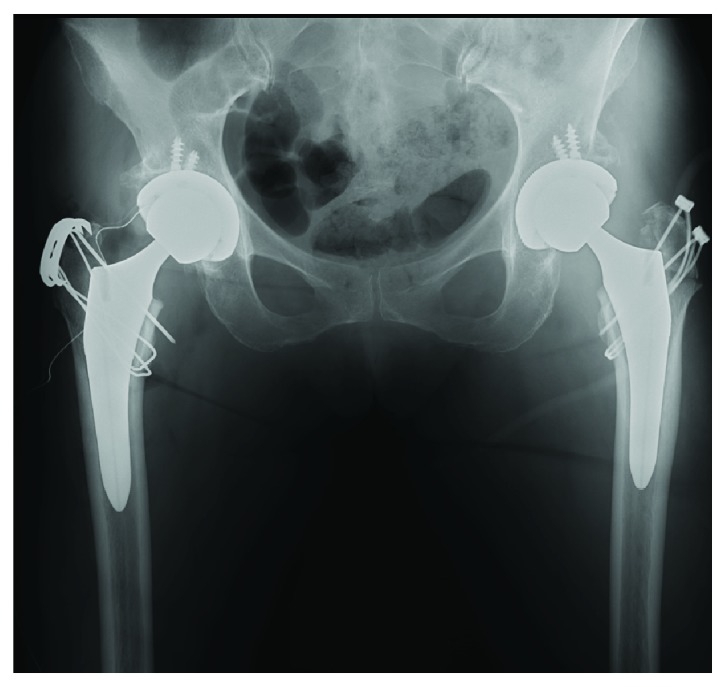
On the 27th postoperative day, the right fracture was treated with small plate and wiring.

Rehabilitation and full weight-bearing exercises were initiated soon after the surgery. The patient was able to perform exercises for walking, muscle strengthening, and range of motion without severe pain. However, on the 14th postoperative day, she complained of left coxalgia during a walking exercise without any falls. Plain radiographs revealed a left greater trochanteric avulsion fracture (Figure 5). This fracture was fixed using tension band wiring on the 18th postoperative day (Figure 6). She was allowed to ambulate but weight bearing of the left leg was prohibited. On the 20th postoperative day, right coxalgia occurred during a transfer exercise, and a greater trochanteric avulsion fracture on the right side was detected on plain radiographs (Figure 7). On the 27th postoperative day, her right fracture was treated with small plate and wiring (Figure 8). The patient was discharged home about a month after the last surgery.

**Figure 9:**
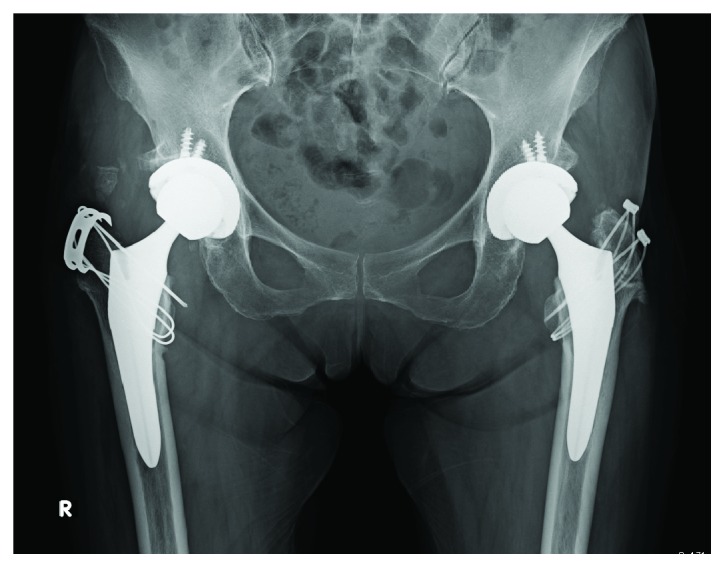
At the 18th postoperative month, the right fragment showed slight upper migration.

In the 18th postoperative month, although the right fragment showed slight upper migration, the patient had no complaints of coxalgia and both hip joints showed excellent range of motion. The Harris Hip Score was 89/89 (Rt/Lt) points with range of motion of 10° bilateral adduction (Figure 9). Informed consent was obtained from the patient to publish this case report. All surgical procedures were conducted in accordance with the Declaration of Helsinki (1964). The report has been approved by the Ethical Committee/Institutional Review Board.

## 3. Discussion

BS-THA was first reported by Jaffe and Charnley in 1971 [8]. Previous reports have showed no difference in systemic complications between 1-stage bilateral THA and 2-stage unilateral THA, and no differences were observed in intraoperative fractures [3–7]. BS-THA was mostly performed either via the direct anterior approach or the posterior approach in previous reports. Greater trochanteric fractures after THA performed via direct anterior approach are not rare and have an incidence of about 12% because the greater trochanter is sometimes subject to excessive load stress due to the surgical procedure of lifting the femur for preparing the stem installation during surgery [1]. Our patient underwent BS-THA via a posterolateral approach and experienced spontaneous bilateral greater trochanteric fractures within the 20th postoperative day but not immediately after the surgery. Among the reports on fractures after BS-THA in the early postoperative phase, although there is a report of a fracture around the femoral stem occurring after BS-THA was performed using an anterior approach, no case of bilateral postoperative femoral large trochanter avulsion fractures after BS-THA with a posterior approach has been reported. Thus, to the best of our knowledge, this case is the first to report such fractures.

Fractures are considered to be associated with multiple factors, such as osteopenia, contracture of the gluteus medius, the height and shape of the femoral neck osteotomy due to the stem design, repairing of the posterior soft tissue, increasing tension of the gluteus medius after operation, and the load of muscular strength training.

With regard to osteopenia, preoperative examination revealed that slight osteopenia was observed in the present case. To date, no reports have demonstrated the relationship between the decrease in BMD and fracture at the greater trochanter and showed frequent fractures at the greater trochanter after THA for elderly people with femoral neck fractures. Moreover, osteopenia is not considered a primary factor for greater trochanter fractures after THA. Osteopenia may have been a minor contributor to the fracture in this case.

Although severe multidirective preoperative hip joint contractures were not found clearly on medical examination, our patient's preoperative abduction was decreased, suggesting the reduction of gluteus medius extensibility.

The femoral stem design used to be one of the causes of greater trochanter fractures. The design of the Charnley cement stem does not usually require the removal of the cancellous bone in the great trochanter area. On the other hand, with the condition wherein the shoulder of the cementless stem is larger and in the valgus femur, the remaining cancellous bone of the greater trochanter becomes very thin and brittle. The remaining bone width of the greater trochanter part in this case was not significantly thinner compared to that after THA using other stems. This is because a tapered wedge-type stem with a design that does not overhand the greater trochanter was used, and the height of femoral neck osteotomy was not too far from the top of the great trochanter, which usually occurs. Moreover, it is difficult to conclude based on the almost horizontal fracture line of our patient that the bone holes that were created vertically in the intertrochanteric posterior crest for the repair of the posterior soft tissue were the main cause. Thus, the kind of stem, osteotomy of the femoral neck fracture, and bone holes are unlikely to be important factors influencing the occurrence of fractures.

It was considered that the spontaneous pelvic coronal tilt toward the surgical side for alleviating the tension of the gluteus muscle, which was often found after unilateral surgery, was difficult to achieve in cases of BS-THA because the pelvis was pulled bilaterally due to the tension of the gluteus muscle, in a manner different from that in unilateral THA. Thus, the tension of the gluteus muscle after BS-THA would be relatively increased when the leg length and offset distance between the pelvis and femur would be longer than those before surgery. Although excessive tension of the affected medial gluteus muscle can be compensated for by abduction of the affected side in unilateral THA, BS-THA cannot prevent the gluteus medius muscle tension at the time of loading. Because the three-dimensional offset of the greater trochanter after operation was longer compared to that before the operation, the tensile strength was more likely to be applied to the attachment part of the greater trochanter gluteus medius muscle, causing fracture at the same part. Because there is no report of a similar case with BS-THA, bilateral great trochanteric fractures after BS-THA in this case are considered to occur due to a complex interaction among several factors.

This report has a limitation in that the cause of the fracture in the present case was not proven with clear evidence. However, to prevent the greater trochanter fracture after BS-THA, surgeons must carefully identify indications in cases with contracture of abduction, small range of motion to adduction, and severe osteoporosis. Additionally, it is necessary to correct the offset such that it is not longer than the preoperative offset.
